# Case Report: A rare case of Noonan syndrome with multiple lentigines manifesting as cardiac enlargement

**DOI:** 10.3389/fcvm.2025.1490436

**Published:** 2025-01-17

**Authors:** Linghua Fan, Jie Jiang, Yan Zhang, Xiaoning Han, Wenhui Ding, Xiaodi Xue, Yimeng Jiang

**Affiliations:** Department of Cardiology, Peking University First Hospital, Beijing, China

**Keywords:** Noonan syndrome with multiple lentigines, heart failure, cardiomegaly, atrial fibrillation, LEOPARD syndrome

## Abstract

Noonan syndrome with multiple lentigines (NSML) is a rare autosomal dominant disorder, primarily caused by variants in the *PTPN11* gene. Characterized by multiple lentigines, hypertelorism, short stature, and hearing loss, its common cardiac manifestations include pulmonary stenosis, hypertrophic cardiomyopathy (HCM), atrial septal defect, and left-sided heart lesions. We report a 58-year-old female diagnosed with NSML presenting with bilateral atrial and ventricular chamber enlargement and atrial fibrillation, which are uncommon cardiac phenotypes of NSML.

## Background

Noonan syndrome with multiple lentigines (NSML), previously known as LEOPARD syndrome, is a rare multisystem autosomal dominant disorder first described in 1969. It is characterized by lentigines, electrocardiographic conduction abnormalities, hypertelorism, pulmonary stenosis, genital anomalies, growth retardation, and deafness (1, 2). Approximately half of the cases are due to variants in the *PTPN11* gene, affecting the RAS-MAPK signaling pathway, which influences cell proliferation, migration, and differentiation (3). Around 85% of patients have cardiac defects, most commonly HCM and pulmonary stenosis, with occasional aortic and mitral valve anomalies (4). The electrocardiogram (ECG) shows not only abnormalities associated with hypertrophic cardiomyopathy but also conduction abnormalities (4). However, the clinical features and genetic heterogeneity of this condition vary significantly among patients (3).

## Case presentation

**Figure 1:**
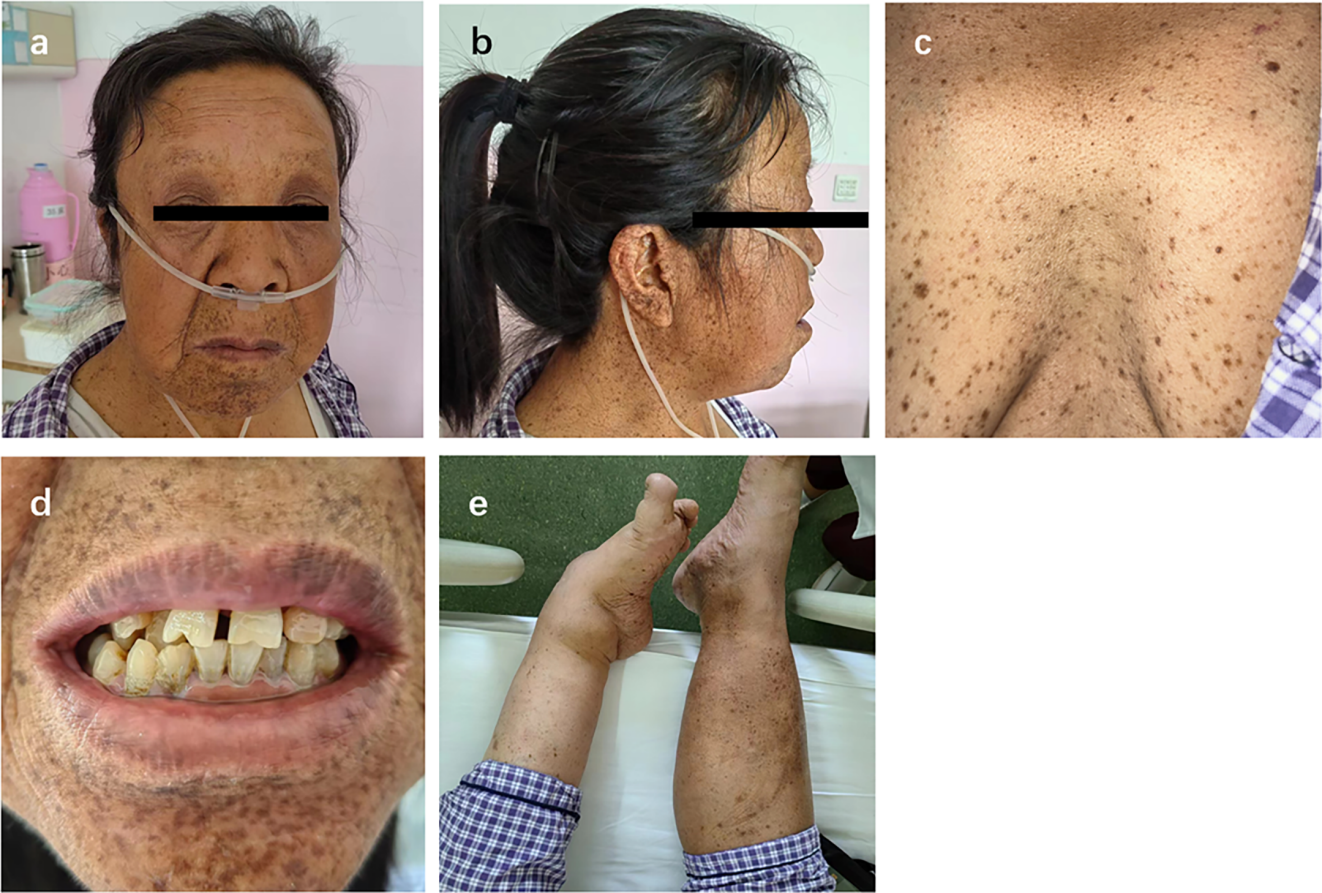
Clinical features of the patient. **(a,b)** Facial characteristics; **(c)** Lentigines and pectus excavatum; **(d)** Facial lentigines and dental malalignment; **(e)** Left foot deformity.

A 58-year-old female presented with a 10-year history of exertional dyspnea, progressively worsening bilateral lower extremity edema, and new-onset orthopnea. She had a history of congenital bilateral hearing loss. Diffuse brown pigmented macules appeared on the face, trunk, and limbs since childhood, with clear borders and varying sizes and shapes. Physical examination revealed short stature (height: 158.5 cm), high forehead hairline, hypertelorism, ptosis, low-set ears, short neck, dental malalignment, and pectus excavatum. Left-sided talipes equinovarus was also noted (Figure 1). Her intellectual development was normal. Cardiac examination showed an enlarged heart, irregular rhythm, and grade 3/6 systolic murmurs at the mitral and tricuspid areas. Jugular venous distention and significant bilateral lower extremity edema were also found. The patient had eight siblings. Through telephone communication and photographic evidence, none of them had a history of heart disease, generalized macules, or developmental anomalies. Her first husband was intellectually disabled, and they had a son who was also intellectually disabled with multiple lentigines. With her second husband, she had a daughter who was physically and intellectually normal and had no lentigines.

**Figure 2:**
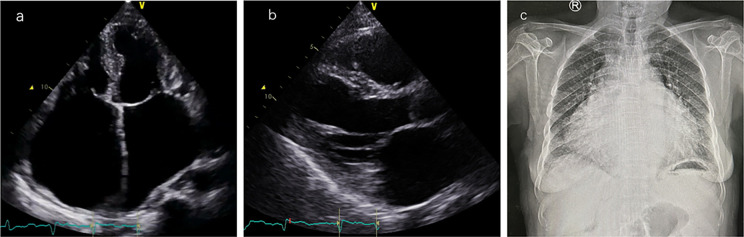
Cardiac imaging findings. **(a)** Apical four-chamber view: the left atrium has a vertical diameter of 8.9 cm and a transverse diameter of 5.9 cm; the right atrium has a vertical diameter of 9.2 cm and a transverse diameter of 6.6 cm. **(b)** Left ventricular long-axis view: left ventricular end-diastolic diameter (LVEDD) is 5.8 cm, left ventricular posterior wall thickness (LVPWT) is 1.1 cm, interventricular septal thickness (IVS) is 1.0 cm, and right ventricular anteroposterior diameter is 2.4 cm. **(c)** Anteroposterior chest x-ray showed significant heart enlargement.

**Figure 3:**
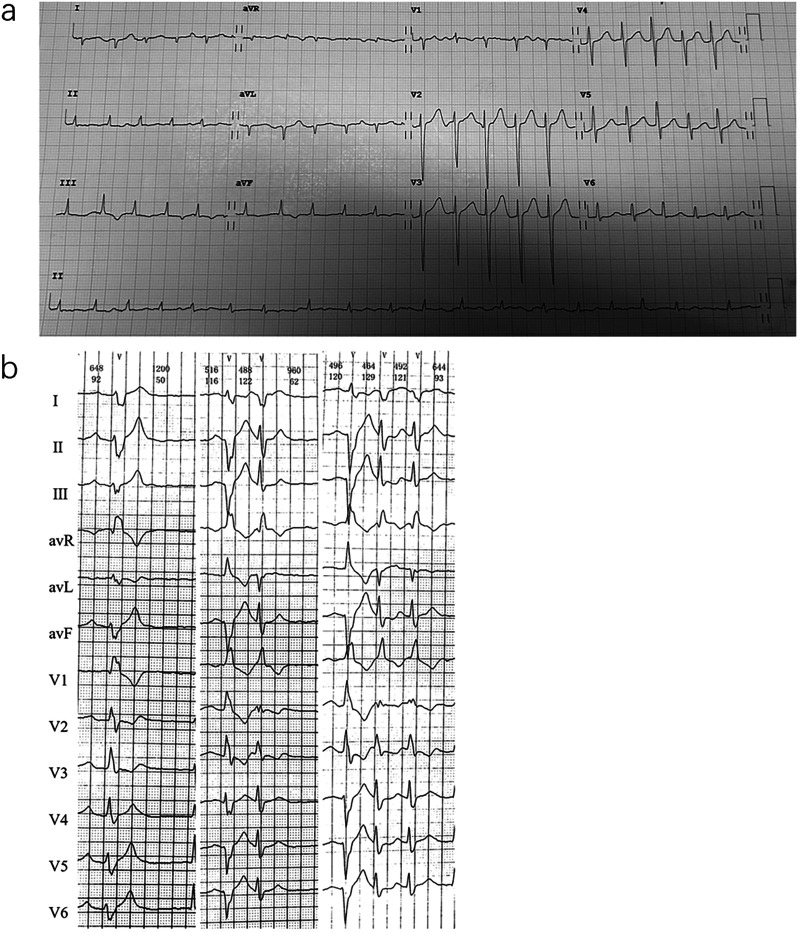
Electrocardiographic findings. **(a)** ECG on admission: atrial fibrillation. **(b)** Holter monitoring: multiple premature ventricular contractions of varying morphologies, some occurring in pairs, and short runs of ventricular tachycardia.

**Figure 4:**
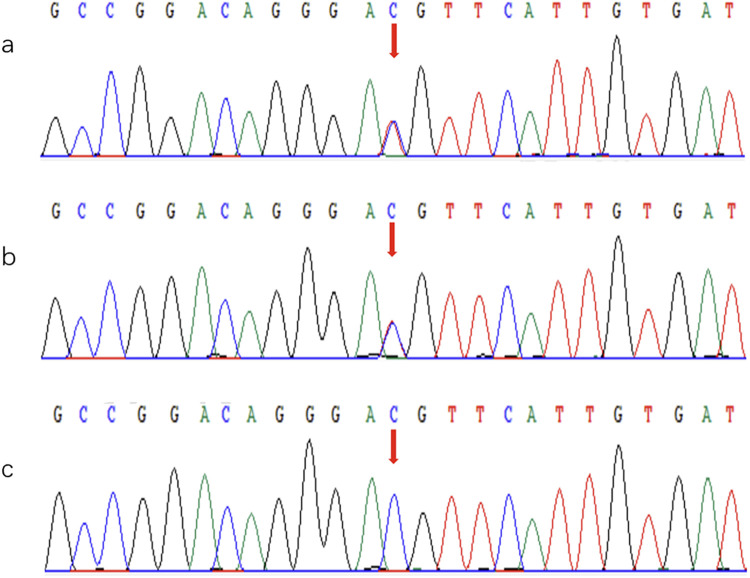
Sanger sequencing of the *PTPN11* variant, exon 12, c.1403 C>T (p.Tyr468Met). **(a)** Proband: Heterozygous mutation detected at the indicated position (red arrow). **(b)** Proband's son: Heterozygous mutation detected at the same position (red arrow). **(c)** Proband's daughter: No mutation detected at this position.

Laboratory tests showed brain natriuretic peptide (BNP) 845 pg/ml and high-sensitivity cardiac troponin I (hs-cTNI) 9.0 ng/L. Echocardiography (UCG) and chest x-ray (Figure 2) revealed global cardiac enlargement (left atrium: anteroposterior diameter 6.4 cm, superior-inferior diameter 8.9 cm, transverse diameter 5.9 cm; right atrium: superior-inferior diameter 9.2 cm, transverse diameter 6.6 cm with an interatrial membrane; left ventricle: end-diastolic diameter 5.8 cm, end-systolic diameter 3.3 cm, posterior wall thickness 1.1 cm, interventricular septum 1.0 cm; right ventricle: anteroposterior diameter 2.4 cm, transverse diameter 5.1 cm), left ventricular ejection fraction 73.7%, severe mitral and tricuspid regurgitation, and an inferior vena cava diameter of 3.6 cm. Septal defect or pulmonary artery stenosis was not found. ECG indicated atrial fibrillation with a ventricular rate of 117 bpm (Figure 3). 1,530 premature ventricular contractions (PVCs) of various morphologies and 4 short runs of ventricular tachycardia were recorded by Holter ECG throughout the day (Figure 3). Furthermore, the patient reported no history of alcohol, drug, or tobacco use, and there was no diagnosis of hypertension. While coronary imaging could not be performed due to the patient's personal preference, bilateral carotid artery ultrasound revealed no atherosclerotic plaques, suggesting a low likelihood of coronary artery disease as a contributing factor. Whole exome sequencing was performed due to suspicion of NSML, revealing a heterozygous pathogenic variant in *PTPN11*, exon 12, c.1403 C>T (p.Tyr468Met) in the blood samples of both the patient and her son (Figure 4). However, this mutation was not detected in her daughter's blood sample (Figure 4). Genetic testing was not performed on her siblings. Other pathogenic genetic alterations related to cardiomyopathy and arrhythmias were not found to have significant relevance, especially genes for the Rasopathies and the cardiovascular phenotype based on the American College of Medical Genetics and Genomics and the Association for Molecular Pathology guidelines (5). The patient was treated with oral anticoagulants, beta-blockers, and diuretics. Symptoms improved, and the patient was discharged with a follow-up plan. The first follow-up visit was conducted after 15 months. The patient's heart rate is well-controlled, with a mean ventricular rate of 79 beats per minute on Holter monitoring, which indicated 24 h of atrial fibrillation and 26 premature ventricular contractions. The left ventricular end-diastolic diameter remained unchanged at 5.6 cm, leading us to conclude that the likelihood of tachycardia-induced cardiomyopathy is low.

## Discussion

In this case report, we highlight the cardiac manifestations of a patient with NSML. Unlike most NSML patients, this patient did not exhibit common cardiovascular manifestations such as hypertrophic cardiomyopathy or pulmonary stenosis. Instead, the primary manifestations were whole cardiac enlargement, heart failure with preserved ejection fraction, and atrial fibrillation. As far as we know, previous NSML cases have not reported such a cardiac phenotype with whole cardiac enlargement (4, 6–8). This case contributes to a broader understanding of the cardiac manifestations of NSML.

NSML is known as Leopard syndrome previously and is classified as a RASopathy, which refers to a group of related disorders caused by variants in genes within the Ras/mitogen-activated protein kinase (Ras/MAPK) pathway (9). The prevalence of NSML is unknown, but it is considered a rarer phenotype among RASopathies. Multiple gene variants can cause Noonan Syndrome/NSML, including *PTPN11*, *BRAS*, and *RAF1*, with *PTPN*11 variants being the most common one. Variants in exon 7 and exon 12 of the *PTPN11* gene, particularly the Tyr279Cys and Thr468Met varitants, are most frequent (10). The cardiac phenotype of patients may be related to specific exon variants. For example, variants in exon 7 and exons 12 and 13 are associated with the septal morphology of hypertrophic cardiomyopathy (11). The protein product of *PTPN11*, the protein tyrosine phosphatase (SHP2), plays a clear role in valve morphogenesis. In mouse models, cardiac defects are caused by the activation of mutant SHP2 in the developing endocardium, which further affects downstream signaling (12). Histologically, myocardial tissues from *PTPN11* mutant mice showed hypertrophy of myocardial cells, disarrayed myocardial fibers, and infiltration of inflammatory cells in the intercellular spaces. As the mice aged to 52 weeks, significant left ventricular hypertrophy transitioned to a dilated cardiomyopathy phenotype, characterized by cardiac enlargement, thinning of the ventricular walls, and impaired cardiac contractility (13) Currently, there is no clear experimental evidence to explain the molecular mechanism underlying the whole cardiac enlargement observed in NSML patients. In one NSML family, elderly patients did not show a cardiac enlargement phenotype (14), but variants in exon 7 of *PTPN11* primarily caused the pathogenic gene in this family. There may still be differences in clinical phenotypes and disease progression among NSML patients with different variants. Besides, current diagnostic tools cannot entirely rule out coronary artery disease or unknown genetic variants that may be contributing to the patient's cardiac enlargement.

In terms of electrophysiological phenotype, multifocal and ectopic atrial tachycardia has been reported in pediatric patients with RASopathies, and some patients respond well to flecainide treatment, suggesting that these patients may have dysregulated calcium ion release, which could lead to cardiomyopathy and heart failure (15). More common ECG findings include ventricular hypertrophy, Q waves, prolonged QT intervals, and repolarization abnormalities (4). There have also been reports of NSML patients exhibiting non-sustained ventricular tachycardia, and it seems that patients without *PTPN11* variants do not have an increased risk of arrhythmia. These patients also have left atrial enlargement, which may be related to diastolic dysfunction or outflow tract obstruction (16). There is no experimental evidence to clarify the mechanism of arrhythmia in NSML patients. We hypothesize that the atrial fibrillation and polymorphic PVCs in this patient may be secondary to cardiac structural changes caused by whole cardiac enlargement.

## Conclusion

We report a patient with distinctive facial features and multiple lentigines who was diagnosed with NSML through genetic testing. The heart is a commonly affected organ in NSML, but the manifestation of whole cardiac enlargement is rare. This case is significant as it emphasizes the importance of recognizing the various cardiac manifestations in NSML, especially in patients with exon 12 of the *PTPN11* mutation, and highlights the need to consider differential diagnoses in heart failure patients with distinctive facial features.

## Data Availability

The raw data supporting the conclusions of this article will be made available by the authors, without undue reservation.

## References

[1] Voron DA Hatfield HH Kalkhoff RK. Multiple lentigines syndrome. Am J Med. (1976) 60(3):447–56. 10.1016/0002-9343(76)90764-6 1258892

[2] Roberts AE Allanson JE Tartaglia M Gelb BD. Noonan syndrome. Lancet. (2013) 381(9863):333–42. 10.1016/S0140-6736(12)61023-X 23312968 PMC4267483

[3] Tartaglia M Gelb BD. Noonan syndrome and related disorders: genetics and pathogenesis. Annu Rev Genomics Hum Genet. (2005) 6:45–68. 10.1146/annurev.genom.6.080604.162305 16124853

[4] Limongelli G Pacileo G Marino B Digilio MC Sarkozy A Elliott P Prevalence and Clinical Significance of Cardiovascular Abnormalities in Patients With the LEOPARD Syndrome. Am J Cardiol. (2007) 100(4):736–41. 10.1016/j.amjcard.2007.03.093 17697839

[5] Richards S Aziz N Bale S Bick D Das S Gastier-Foster J Standards and guidelines for the interpretation of sequence variants: a joint consensus recommendation of the American College of Medical Genetics and Genomics and the Association for Molecular Pathology. Genet Med. (2015) 17(5):405–24. 10.1038/gim.2015.30 25741868 PMC4544753

[6] Delogu AB Limongelli G Versacci P Adorisio R Kaski JP Blandino R The heart in RASopathies. Am J Med Genet Part C. (2022) 190(4):440–51. 10.1002/ajmg.c.32014 36408797

[7] Hilal N Chen Z Chen MH Choudhury S. RASopathies and cardiac manifestations. Front Cardiovasc Med. (2023) 10:1176828. 10.3389/fcvm.2023.1176828 37529712 PMC10387527

[8] Baldo F Fachin A Da Re B Rubinato E Bobbo M Barbi E. New insights on Noonan syndrome’s clinical phenotype: a single center retrospective study. BMC Pediatr. (2022) 22(1):734. 10.1186/s12887-022-03804-2 36566191 PMC9789552

[9] Feng G-S Pawson T. Phosphotyrosine phosphatases with SH2 domains: regulators of signal transduction. Trends Genet. (1994) 10(2):54–8. 10.1016/0168-9525(94)90149-X 8191586

[10] Sarkozy A Conti E Seripa D Digilio MC Grifone N Tandoi C Correlation between PTPN11 gene mutations and congenital heart defects in Noonan and LEOPARD syndromes. J Med Genet. (2003) 40(9):704–8. 10.1136/jmg.40.9.704 12960218 PMC1735592

[11] Kauffman H Ahrens-Nicklas RC Calderon-Anyosa RJC Ritter AL Lin KY Rossano JW Genotype-phenotype association by echocardiography offers incremental value in patients with Noonan syndrome with multiple lentigines. Pediatr Res. (2021) 90(2):444–51. 10.1038/s41390-020-01292-7 33318624

[12] Araki T Chan G Newbigging S Morikawa L Bronson RT Neel BG. Noonan syndrome cardiac defects are caused by *PTPN11* acting in endocardium to enhance endocardial-mesenchymal transformation. Proc Natl Acad Sci U S A. (2009) 106(12):4736–41. 10.1073/pnas.0810053106 19251646 PMC2649209

[13] Marin TM Keith K Davies B Conner DA Guha P Kalaitzidis D Rapamycin reverses hypertrophic cardiomyopathy in a mouse model of LEOPARD syndrome–associated PTPN11 mutation. J Clin Invest. (2011) 121(3):1026–43. 10.1172/JCI44972 21339643 PMC3049377

[14] Chan C-H Chu M-F Lam UP Mok T-M Tam W-C Tomlinson B Case report: Distinctive cardiac features and phenotypic characteristics of Noonan syndrome with multiple lentigines among three generations in one family. Front Cardiovasc Med. (2023) 10:1225667. 10.3389/fcvm.2023.1225667 37692036 PMC10484218

[15] Levin MD Saitta SC Gripp KW Wenger TL Ganesh J Kalish JM Nonreentrant atrial tachycardia occurs independently of hypertrophic cardiomyopathy in RASopathy patients. Am J Med Genet A. (2018) 176(8):1711–22. 10.1002/ajmg.a.38854 30055033 PMC6107379

[16] Limongelli G Sarkozy A Pacileo G Calabrò P Digilio MC Maddaloni V Genotype–phenotype analysis and natural history of left ventricular hypertrophy in LEOPARD syndrome. Am J Med Genet Part A. (2008) 146A(5):620–8. 10.1002/ajmg.a.32206 18241070

